# Smart home energy management for sustainable socioeconomic development in Egyptian households

**DOI:** 10.1038/s41598-026-35705-0

**Published:** 2026-02-06

**Authors:** Omar Saif, Rasha Elazab, Mohamed Daowd

**Affiliations:** Faculty of Engineering at Electrical, Power and Machines department, Capital University (Formerly: Helwan University), Cairo, Egypt

**Keywords:** Smart home energy management system, Inclining block rate tariff, Load scheduling, Photovoltaic system, Battery storage, Developing countries, Energy and society, Energy management

## Abstract

Smart Home Energy Management Systems (SHEMS) play a critical role in reducing household energy consumption. However, existing solutions often depend on complex infrastructure and dynamic pricing models, limiting their applicability in developing countries like Egypt, where Inclining Block Rate (IBR) tariffs are in effect. This study presents a low-cost, hardware-minimal SHEMS prototype designed for Egypt’s socio-economic and climatic context. The system incorporates a comfort zone-based load scheduling algorithm, which allows users to define acceptable used area for according to its facilities and input the number of active household members over three timeframes (hourly, daily, weekly). Based on this input, the system recommends selective air-conditioner usage to minimize costs without compromising comfort, while leaving the final decision to the user. This approach promotes energy awareness and preserves user autonomy. The prototype integrates photovoltaic (PV) generation and battery storage. Results show that the comfort zone strategy yielded the highest annual savings and fastest payback, while the PV-battery system offered the best long-term return. Environmentally, Greater Cairo scale deployment could reduce CO₂ emissions by over 1.4 million ton annually. The system aligns with SDG 7 (Affordable and Clean Energy) and SDG 13 (Climate Action), offering a scalable, context-sensitive solution for sustainable energy management in resource-constrained settings.

**Supplementary Information:**

The online version contains supplementary material available at 10.1038/s41598-026-35705-0.

## Introduction

### Background

SHEMSs aim to optimize appliance operation within households by minimizing energy costs through advanced load scheduling techniques. Traditional scheduling algorithms in SHEMS generally focus on dynamic pricing mechanisms, such as Time-of-Use (TOU) and Real-Time Pricing ((HVAC), widely adopted in developed nations^1–3^. These systems often consider grid tariffs, renewable energy integration, and multiple user objectives^4–6^. However, in developing countries like Egypt, where IBR tariffs dominate, such models often fall short of addressing region-specific challenges. The IBR tariff structure, based on total monthly consumption rather than specific time-of-use rates, makes conventional load-shifting strategies ineffective^8,9^. This necessitates a rethinking of SHEMS to adapt to the socioeconomic realities and energy pricing structures of Egypt.

### Challenges in egypt’s energy landscape

Egypt is facing severe energy challenges due to rapid population growth, reaching 104.4 million in December 2022 and continuing to rise by 1.6 million annually^10^. Despite efforts to expand generation capacity, electricity demand driven by economic growth has strained the power grid. Summer outages remain frequent due to fuel shortages, and fossil fuels account for 89% of the electricity generation mix, leaving only 11% for renewables^11^. These factors, alongside the rising costs of electricity following subsidy reforms, underscore the urgent need for more efficient household energy solutions. This is particularly critical as the country seeks to balance energy needs with sustainability goals.

### Existing approaches and limitations

While there has been a growing body of research on SHEMS in both developed and developing contexts, few studies address the specific challenges posed by IBR tariffs in Egypt. Some attempts have integrated SHEMS with PV systems and optimization techniques like Mixed-Integer Linear Programming (MILP)^12,13^, yet these models often fail to fully consider the socioeconomic factors influencing Egyptian households. Under IBR tariffs, shifting energy consumption throughout the day does not yield significant savings, as charges are based on total monthly consumption. Thus, many of the strategies that work well in developed countries, like dynamic pricing and time-of-use optimizations, are less effective in Egypt’s fixed-rate system.

Additionally, many commercially available SHEMS, such as those from SPAN Panel, Honeywell, and Schneider Electric, are designed for environments with dynamic pricing, and often require advanced sensors and continuous data monitoring. These systems, while effective in high-income regions, are ill-suited to the financial constraints and cultural context of developing countries, where affordability, simplicity, and low user engagement are key considerations.

### Proposed solution: a low-cost, culturally aligned prototype

**Fig. 1:**
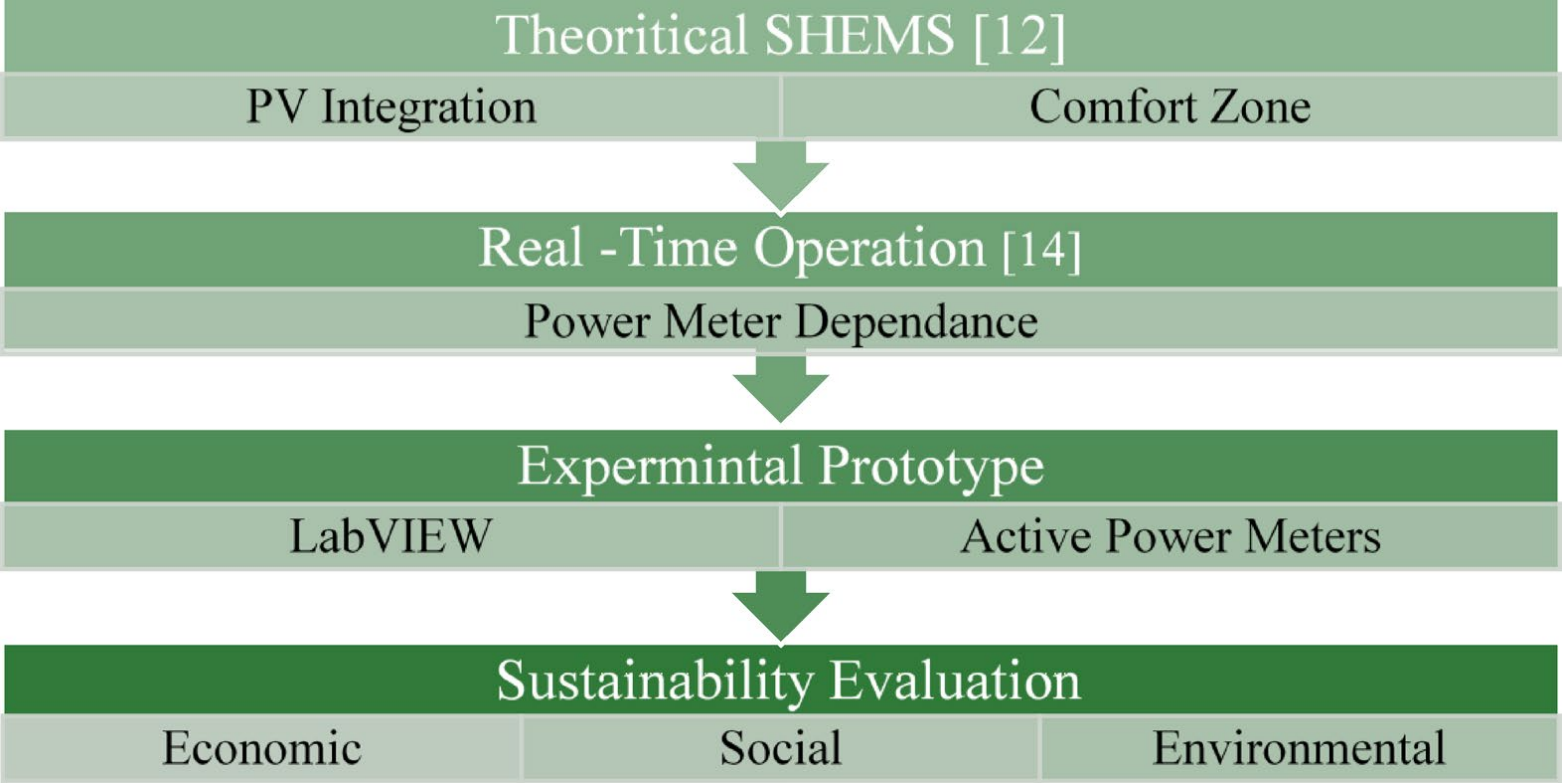
Developmental framework of the proposed SHEMS.

To address these gaps, this study proposes a low-cost, minimal-hardware SHEMS prototype tailored for Egyptian households under IBR tariffs. The system features a novel “comfort zone” based load scheduling approach, designed to optimize energy use and reduce costs without requiring extensive hardware upgrades or sophisticated sensors^12^. proposed theoretical energy-saving models for Egypt by authors, but our approach differs by translating these models into a tangible, practical solution for real-world implementation, see Fig. 1. The prototype is designed to be simple, affordable, and culturally appropriate, ensuring that it is both accessible and effective in the local context.

This study evaluates the system’s performance across three pillars: economic, social, and environmental. Economic feasibility is assessed through payback periods for three configurations: PV-only, PV-battery, and comfort zone optimization. The social impact is quantified in terms of household-level energy savings and the broader adoption potential, while the environmental benefits are measured by avoided CO₂ emissions.

### Contributions of this study

This study offers several novel contributions:

- Development of a Minimal-Hardware SHEMS Prototype: The study introduces a low-cost SHEMS prototype that operates effectively under IBR tariffs, addressing the unique challenges of Egyptian households without requiring expensive hardware upgrades.
- Comprehensive Sustainability Evaluation: A key contribution is the integrated sustainability evaluation, which quantifies the economic feasibility, societal impacts, and environmental benefits of adopting the proposed system.
- Comfort zone-based AC scheduling: Based on user-defined occupant numbers across hourly, daily, and weekly timeframes, the system suggests which AC units can be turned off with minimal comfort impact.
- Estimated cost savings: For each configuration, the system provides projected electricity bill reductions, allowing users to assess potential benefits.
- User-driven decision-making: Final control is left to the homeowner, promoting awareness, preserving autonomy, and ensuring satisfaction in energy-saving participation.

### Problem statement and study scope

The problem addressed in this study is the lack of affordable, effective SHEMS solutions for Egyptian households, where IBR tariffs dominate and affordability is a primary concern. Conventional SHEMS, designed for dynamic pricing environments, fail to meet the needs of Egyptian consumers. This study proposes an alternative approach, a low-cost, user-friendly SHEMS prototype that aligns with the local energy pricing structure and socioeconomic conditions.

By developing a practical prototype and assessing its impacts on energy use, this study aims to fill a critical gap in existing literature, offering a unique contribution to SHEMS research. The scope of the study is focused on the evaluation of economic, social, and environmental impacts of this prototype.

### Motivation and novelty

The motivation behind this study is to provide a practical solution for improving household energy efficiency in Egypt while considering the unique challenges posed by IBR tariffs. The novelty of this work lies in the development of a cost-effective SHEMS prototype that adapts to the local tariff structure, providing a viable alternative to systems based on dynamic pricing. Additionally, the introduction of a comfort zone-based scheduling approach is a key innovation, as it allows for significant energy savings without requiring user behavior changes or advanced infrastructure.

This study contributes to the growing body of SHEMS research by offering a solution that is both technically innovative and culturally aligned with the needs of households in Egypt, thereby offering a model that could be replicated in other developing countries with similar energy constraints.

## Methods

### Theoretical proposed scheme

The proposed SHEMS offers a comprehensive framework for optimizing energy use in Egyptian households while maintaining user comfort and minimizing reliance on the grid. This system is implemented in LabVIEW and structured around a three-tiered control hierarchy, hourly, daily, and weekly, to integrate immediate responsiveness with long-term energy planning.

This methodology aims to maximize self-consumption of rooftop PV generation and optimize adjustable loads (primarily HVAC systems) using a comfort zone approach, rather than relying on traditional load-shifting strategies that are ineffective under Egypt’s IBR tariff.

Timeframe-Based Control Logic:

- Hourly Control: Executes real-time decisions based on current PV output, sensor data, and load demand. Adjust HVAC and lighting systems within user-defined comfort zones.
- Daily Planning: Forecasts solar availability and user occupancy patterns to optimize battery charging/discharging and manage grid reliance for the coming day.
- Weekly Planning: Uses broader consumption and generation trends to set baseline strategies for battery reserve, grid use, and thermal regulation.

Key operational strategies:

- PV Self-Consumption Optimization: Prioritizes using PV energy for immediate loads and charging batteries when generation exceeds demand.
- Battery Management: Maintains the State of Charge (SOC) within a safe range (typically 20–90%) while smoothing load fluctuations.
- Comfort Zone Control: HVAC operation is dynamically adjusted based on the number of users entered manually in the system’s interface across weekly, daily, and hourly timeframes. The system suggests HVAC units switching off when possible and displays the estimated savings, leaving the final decision to the user to ensure comfort and awareness.

**Fig. 2:**
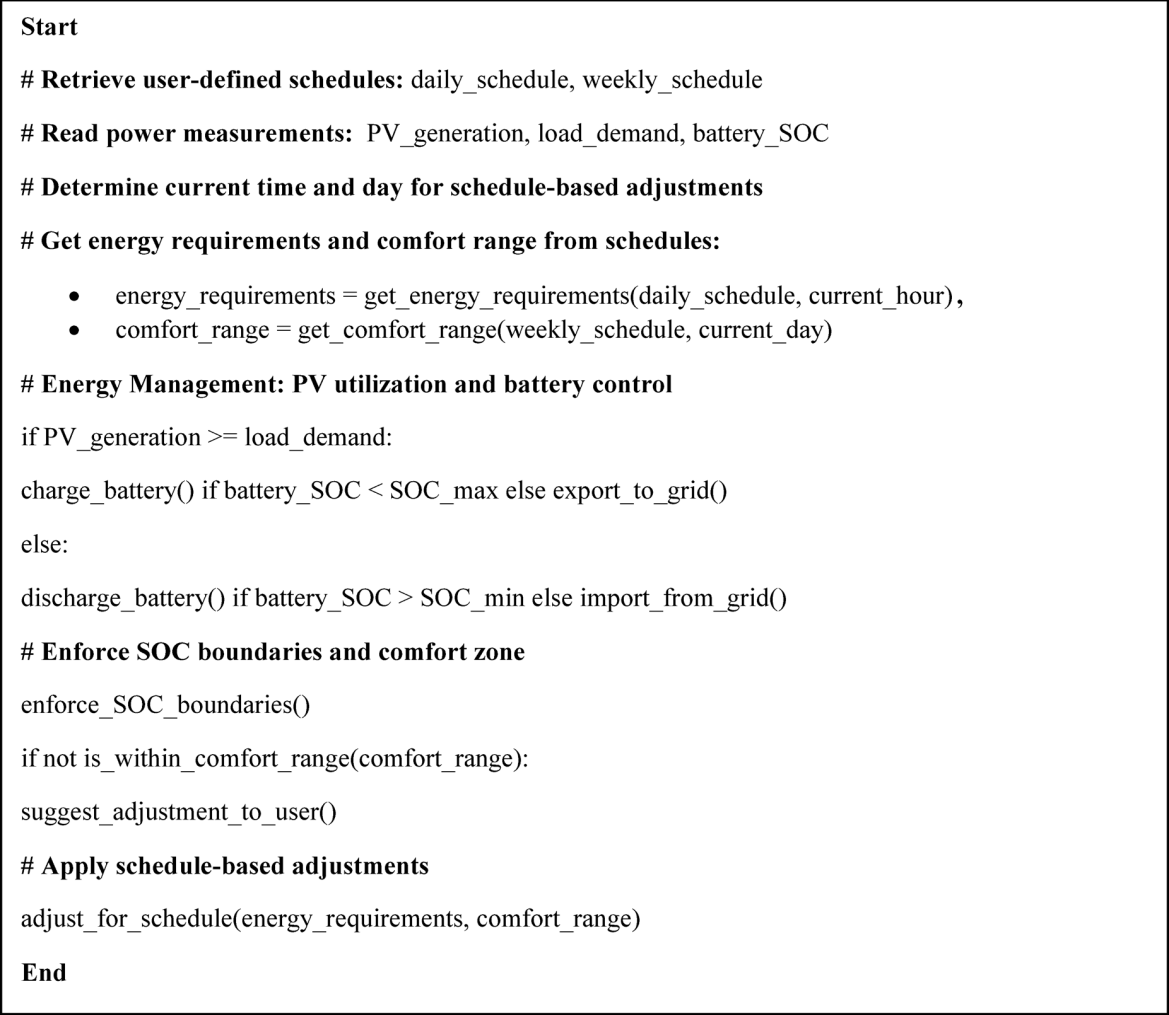
The proposed SHEMS.

This methodology was originally conceptualized and validated theoretically in the authors’ previous work^12^, as shown in Fig. 2. Detailed parameters and simulation models from the theoretical study are available in the supplementary materials. In this paper, the focus shifts to the hardware implementation and real-world testing of the proposed SHEMS. While experimental tests validate the functionality of the control framework and provide actual hardware cost data, the economic evaluation of operational performance relies on extrapolated results from the prior theoretical study due to laboratory limitations such as constrained programmable load availability and dependence on actual solar irradiance. This ensures that sustainability metrics are grounded in robust long-term simulations, while prototype implementation provides both feasibility validation and practical cost data for system components.

### Real time operation

The proposed approach for short-term PV power prediction in the hourly timeframe of SHEMS is designed to be simple, cost-effective, and privacy conscious. Unlike conventional artificial intelligence (AI)-based prediction models, which rely on extensive historical data, complex computations, and high-speed internet connectivity, the proposed method depends solely on real-time PV power measurements and Geographic Information System (GIS) solar radiation data^14^. By leveraging these easily accessible data sources, this approach eliminates the need for expensive sensors and sophisticated learning models, making it particularly suitable for implementation in developing countries.

The proposed methodology relies on the fundamental relationship between measured PV power output and clear/cloudy sky radiation models obtained from GIS data. The algorithm uses the following key steps:

1. GIS-Based Solar Radiation Data: Free solar radiation datasets provide average daily solar profiles under clear and cloudy sky conditions for a given location.
2. Real-Time PV Power Measurement: The actual power output of the PV system is continuously measured using a smart meter.
3. Cloudiness Adaptation Factor: By comparing the measured PV power with the expected power from clear/cloudy sky conditions, an adaptive cloudiness index is calculated.
4. Prediction of Future PV Power: The cloudiness index is used to estimate the next time interval’s solar radiation and, consequently, the expected PV power generation.

**Fig. 3:**
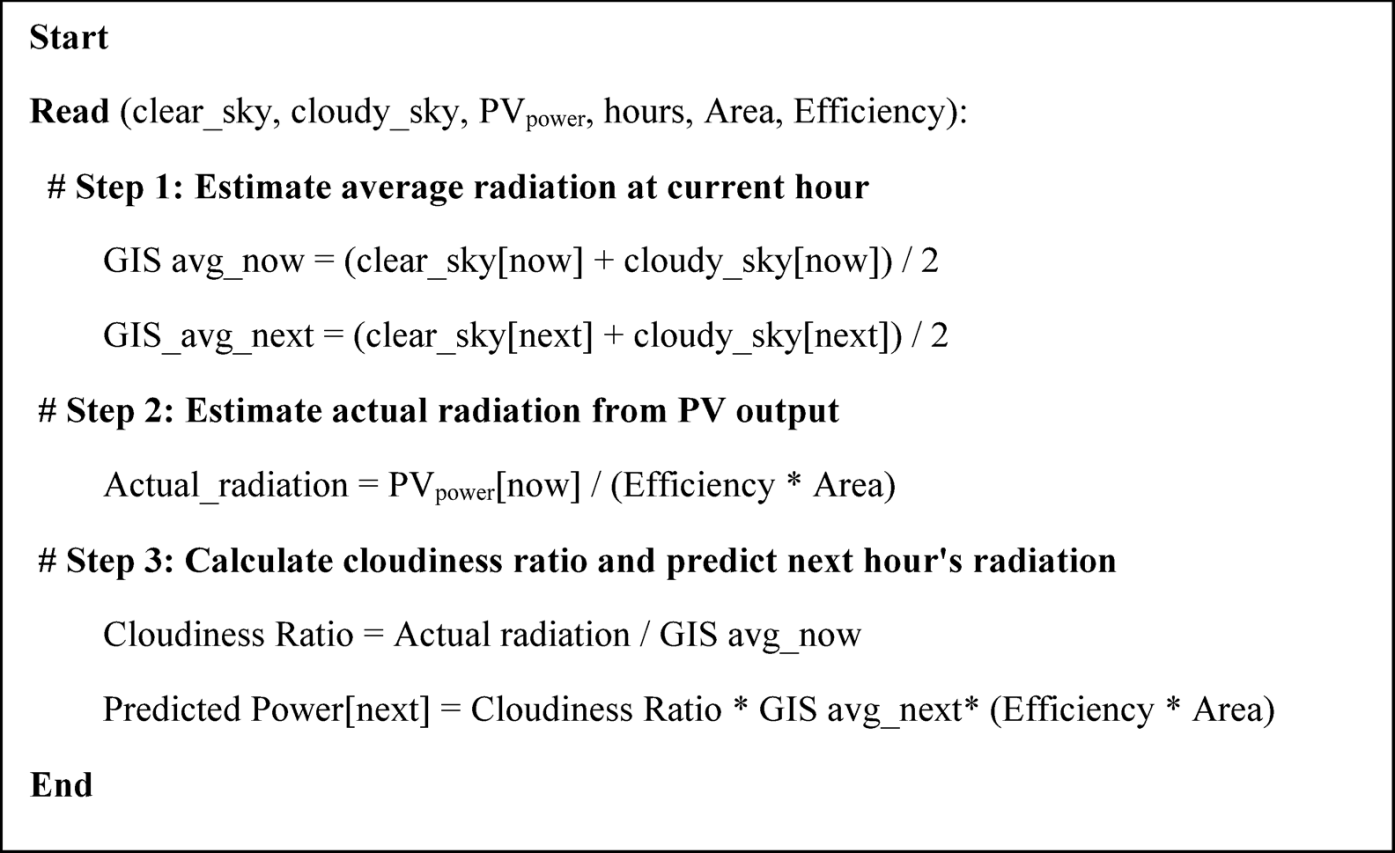
Hourly PV power prediction pseudo code.

This reverse engineering method effectively utilizes available GIS models and real-time PV measurements to achieve accurate and fast short-term predictions without requiring long-term historical data analysis or computationally intensive training models, as explained in Fig. 3.

**Fig. 4:**
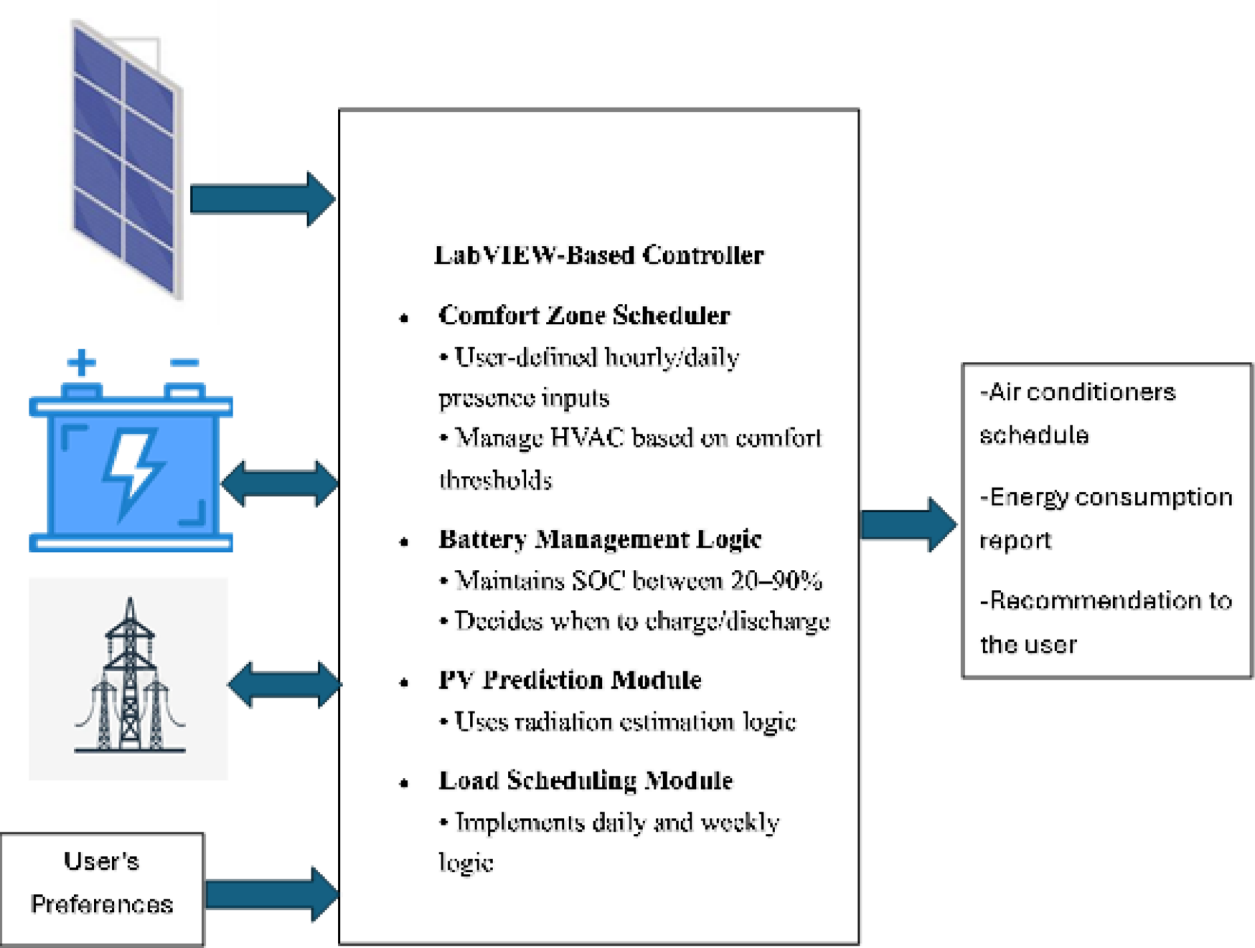
SHEMS block diagram.

Unlike cloud-based AI models that require users to share their consumption and energy data with third parties, the proposed method operates locally, ensuring complete data privacy. The model only requires a meter for power measurements and a pre-loaded GIS database, making it cost-effective and feasible for widespread adoption. Since it does not involve complex neural networks or AI training processes, the method can be deployed on low-cost microcontrollers, reducing hardware and operational costs^14^. Figure 4 shows hardware-software interaction of the proposed SHEMS.

### Experimental setup

**Fig. 5:**
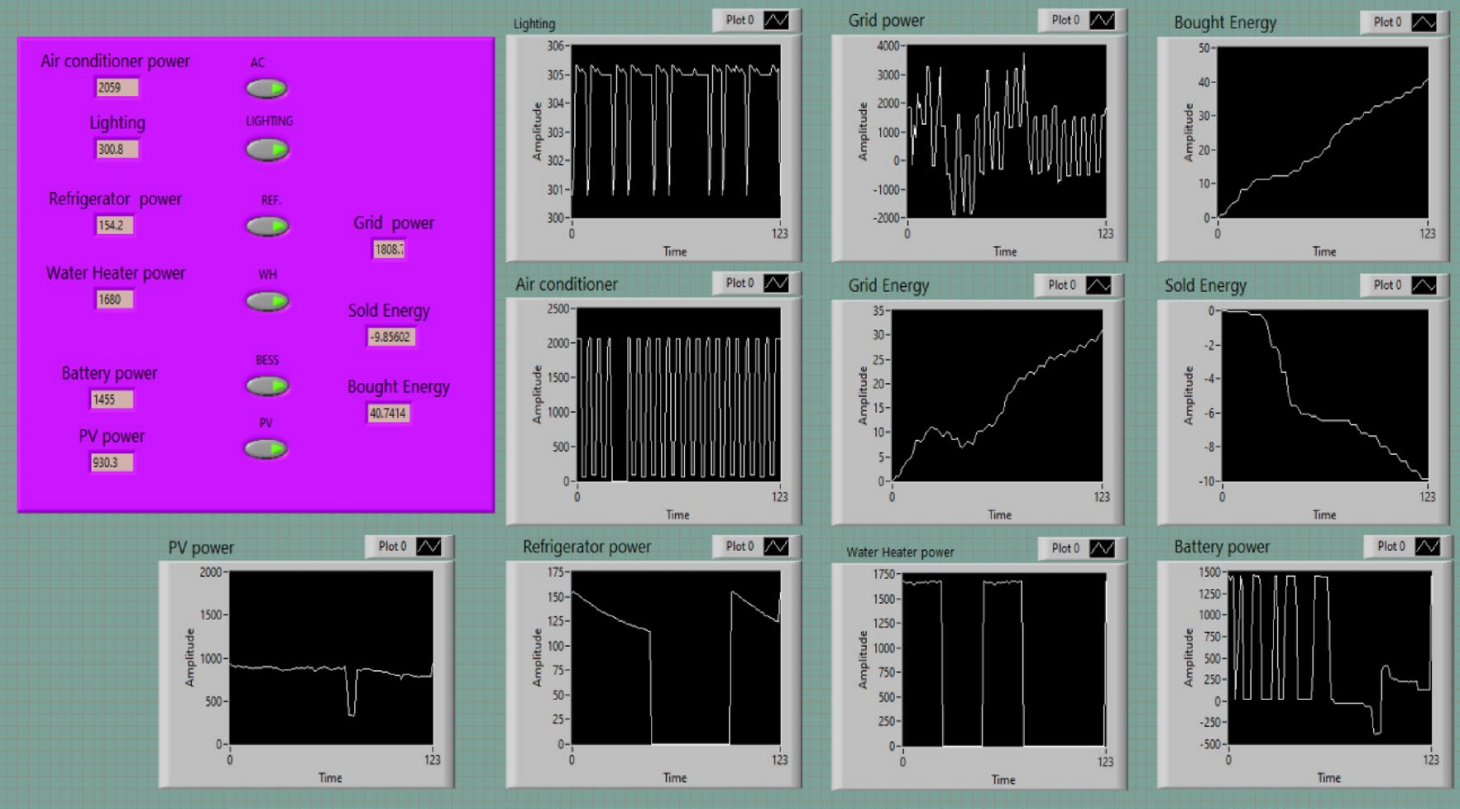
The user interface screen.

The experimental setup featured a User Interface Screen (UIS) developed using LabVIEW software, serving as the primary hub for monitoring and controlling SHEMS, see Fig. 5. This interface provided real-time data visualization, including instantaneous power consumption of appliances and energy flows between the grid, PV system, and battery. Key metrics such as grid power, PV power, and battery power were dynamically displayed, along with graphical plots of energy trends, enabling comprehensive system performance analysis under varying conditions.

A major highlight of the UIS was its appliance-specific monitoring, which allowed detailed tracking of devices like air conditioners, refrigerators, and water heaters. This feature facilitated the identification of high-energy-consuming appliances and validated energy-saving strategies, such as targeted cooling during comfort zone management scenarios. The interface also visually represented energy flows, showing how PV-generated energy was distributed among the battery, household loads, or sold to the grid, and tracking grid energy usage to minimize reliance on external sources.

The interface supported interactivity, allowing users to control appliance operations in real time, adjust schedules, and set comfort zone parameters. It also enabled historical data analysis, providing insights into the long-term effectiveness of SHEMS strategies. By bridging the gap between theoretical models and practical implementation, the UIS validated the feasibility and effectiveness of the proposed energy management system, particularly under IBR tariffs. This intuitive and integrated platform highlighted the potential of SHEMS to optimize energy savings and reduce costs for households.

While LabVIEW served as an effective prototyping tool for proof-of-concept validation due to its powerful interface and National Instruments DAC 6002 compatibility, we recognize that it is licensed commercial software and may not be suitable for large-scale consumer deployment.

Therefore, we propose that open-source alternatives such as Python (with libraries like Dash/Plotly), Home Assistant, or Node-RED can replace LabVIEW in the final consumer-ready implementation. These platforms support integration with microcontrollers (e.g., Raspberry Pi, Arduino) and allow cost-effective, scalable deployment.

#### Prototype description

The experimental testbed was meticulously designed to replicate the theoretical models proposed for a SHEMS under an IBR tariff structure. The prototype was developed at Helwan University, integrating various components to emulate the energy dynamics of a typical residential setup. The experimental prototype incorporates an 8 kW rooftop PV system composed of 20 monocrystalline panels rated at 400 W each, installed at optimal tilt and azimuth angles for Cairo’s solar profile. A hybrid inverter (Growatt SPF 5000 ES) is used to interface the PV array, grid, and battery bank. The energy storage system consists of a 30 kWh lithium-ion battery bank with a nominal voltage of 48 V and a rated discharge power of 5 kW. The system is managed with a control logic that maintains battery operation between 20% and 90% SOC to ensure both safety and longevity. The control strategy prioritizes PV utilization for load supply and battery charging. During PV surplus hours, the battery charges until it reaches the upper SOC limit. In low PV generation periods or during grid outages, the battery discharges to support the loads but is restricted from discharging below 20% SOC. If both PV and battery are insufficient, the grid supplies the deficit. This rule-based logic is implemented in LabVIEW and executed in real-time via a National Instruments DAC 6002 data acquisition and control unit. The algorithm also ensures that high-priority loads are supplied preferentially in case of limited energy availability.

The prototype also included a variety of electrical appliances representative of a standard Egyptian home. These comprised air conditioners with capacities ranging from 3 to 6 HP for cooling, alongside other typical household devices such as refrigerators, and electric ovens. The control and monitoring of the testbed relied on advanced hardware and software integration. A National Instruments DAC 6002 Controller served as the primary data acquisition unit, enabling precise measurement of power flows and real-time control of the system.

**Fig. 6:**
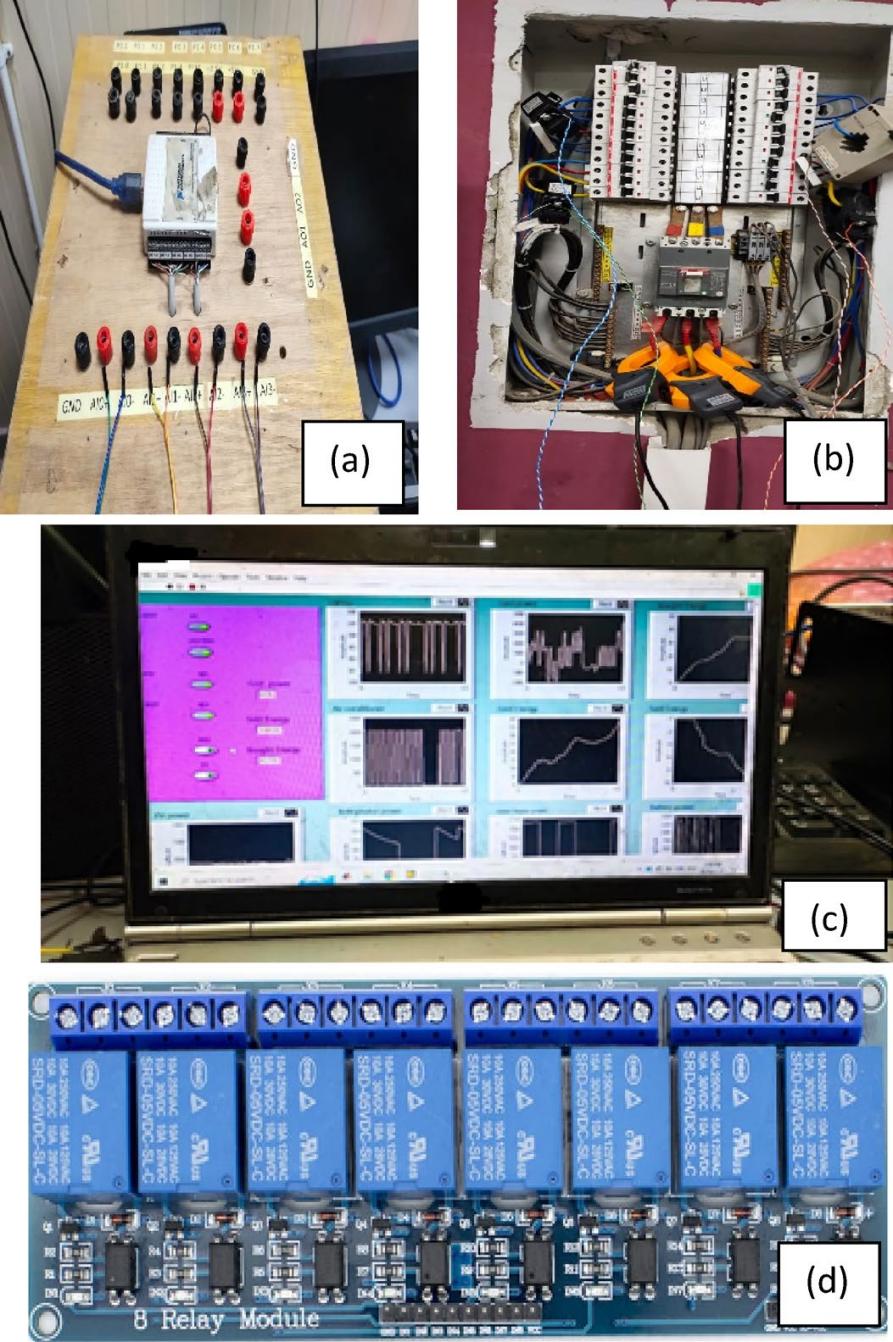
Experimental setup, (**a**) DAC 6002 controller, (**b**) Measurement and breakers, (**c**) UIS, d-Relays modules.

The data acquisition process was further enhanced by LabVIEW software, which provided a user-friendly interface for monitoring and managing the system. This interface not only displayed real-time data but also facilitated adjustments to appliance schedules and system settings, making it a vital component of the experimental framework. The overall design of the prototype ensured that the experimental scenarios were closely aligned with theoretical simulations, providing a robust platform for validating the proposed SHEMS strategies, as shown in Fig. 6.

#### Data collection and measurement parameters

The experimental setup was equipped to collect comprehensive data across various parameters, enabling a thorough evaluation of the SHEMS strategies. Key energy metrics, including PV generation, battery performance, and grid interactions, were continuously monitored and recorded. The PV system’s energy output was measured on an hourly and daily basis, capturing fluctuations in solar radiation and their impact on energy availability. PV power is predicted and managed through the PV power measurement and solar radiation GIS based model, as proposed on^14^. Similarly, the battery’s charging and discharging cycles were tracked, along with SOC variations, to assess the effectiveness of storage utilization in meeting household demands.

Grid power usage was another critical parameter, encompassing both energies consumed from and sold back to the grid. These measurements were essential for calculating the net energy balance and evaluating the financial benefits of each scenario under the IBR tariff structure. Appliance-specific energy consumption was also recorded, providing insights into the operational efficiency of individual devices. This data was particularly important for analyzing the impact of load-shifting and comfort zone management strategies on overall energy use.

In addition to energy metrics, the study incorporated a detailed cost analysis that combined insights from both the experimental prototype and the validated theoretical model. The prototype provided real hardware cost data and demonstrated the feasibility of real-time control under actual operating conditions. However, due to experimental limitations, namely the restricted programmable load and variability of solar irradiance, the prototype alone could not provide long-term operational data or reliable payback metrics. Therefore, monthly energy costs, savings under the IBR tariff, and sustainability indicators (economic, environmental, and social) were derived from the validated theoretical model reported in our earlier work^12^. In this way, the experimental platform ensured hardware realism and control validation, while the theoretical model supplied the long-term performance metrics required for sustainability evaluation. This dual approach maintains both practical validation and analytical completeness.

###  Tariff structure integration

The Egyptian residential electricity pricing follows an IBR system, where consumption is divided into categories, and incremental tariffs are applied progressively according to total monthly usage. If consumption falls in a higher category, all the corresponding block rates of that category are applied, rather than the lower-tier ones. Table 1 summarizes the IBR structure as of 2024^15^.

For example:

- A household consuming 80 kWh/month is billed 50 kWh at 0.68 EGP/kWh and 30 kWh at 0.78 EGP/kWh.
- A household consuming 400 kWh/month falls in the 101–650 kWh category: the first 200 kWh are billed at 0.95 EGP/kWh, the next 150 kWh at 1.55 EGP/kWh, and the remaining 50 kWh at 1.95 EGP/kWh.

**Table 1:** Egyptian residential tariff.

Category	Total monthly consumption (kWh)	Incremental tariff breakdown (EGP/kWh)
**1**	0–100	0–50 at 0.68; 51–100 at 0.78
**2**	101–650	0–200 at 0.95; 201–350 at 1.55; 351–650 at 1.95
**3**	651–1000	651–1000 at 2.10
**4**	> 1000	> 1000 at 2.23

In addition, since excess rooftop PV generation can be exported to the grid under Egypt’s net-metering and feed-in tariff schemes, the current rate of 0.06685 USD/kWh^12^ in the economic analysis. included This ensures transparency in the cost savings and payback period calculations presented in Subsection 3.6.

### Experimental scenarios and energy transactions

To evaluate the effectiveness of the proposed smart home prototype, four distinct operating scenarios were implemented. These scenarios demonstrate the system’s ability to manage energy transactions among the grid, photovoltaic (PV) panels, and battery storage to optimize energy use and economic performance. Each scenario was tested using the LabVIEW platform over a fixed 10-minute window to observe real-time energy flows and quantify energy consumption and grid interaction.

- Scenario 1: Default Case (Grid-Only Operation):
  This baseline scenario represents traditional household operation where all appliances are powered solely by the utility grid. It reflects the absence of any renewable energy integration or energy management control.
- Scenario 2: Grid with PV Integration:
  PV panels are added to the system, enabling the generation of solar energy. Excess energy is sold to the grid at low feed-in tariffs. This setup allows partial self-consumption and revenue generation from solar energy.
- Scenario 3: Grid with PV and Battery Storage:
  A battery is incorporated to store surplus PV energy rather than exporting it to the grid. The stored energy is later used to power household appliances, reducing grid reliance and enhancing energy efficiency.
- Scenario 4: Smart Home Energy Management System:
  This scenario integrates PV panels, a battery system, and an intelligent scheduling algorithm. The SHEMS optimizes appliance usage based on comfort zones and PV availability, thereby reducing peak load, minimizing energy costs, and increasing grid export revenue.
  These scenarios are designed to demonstrate performance progression from a conventional setup to a fully optimized smart home energy management system.

## Results and discussion

The experimental results demonstrated a strong correlation with the theoretical predictions, validating the effectiveness of the proposed SHEMS strategies. Comfort zone management emerged as the most effective strategy in terms of cost savings, with experimental data highlighting the significant impact of selective cooling on overall energy consumption, as discussed in^12^. By reducing air conditioner usage in non-occupied rooms, this strategy achieved energy savings comparable to those predicted in the theoretical simulations. Different power/energy behaviors of SHEMS are tested experimentally, as will be discussed in the following subsections.

The figures presented in this section illustrate the real-time dynamic performance of household appliances under the implemented smart control system. Data were collected using the LabVIEW platform, and each graph covers a time window of 123 s. This short interval was intentionally selected to capture the transient behavior of appliances during switching events and load adjustment, providing insights into the effectiveness of the proposed scheduling algorithm.

### The air-conditioner

**Fig. 7:**
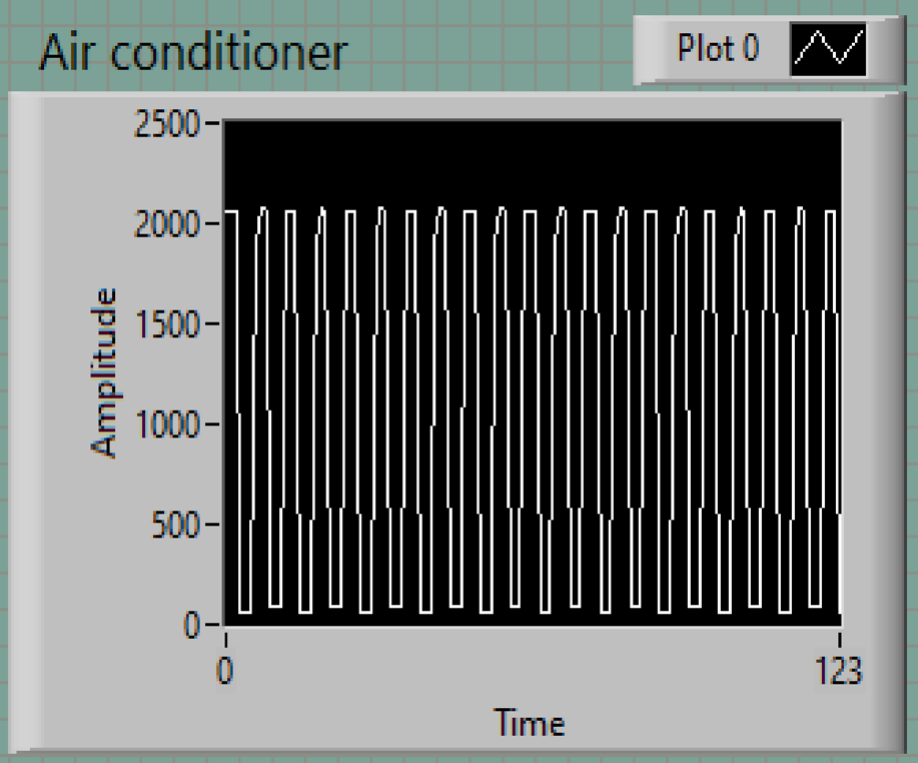
Air-conditioner power.

The power consumption profile of an air conditioner, as shown in Fig. 7, demonstrates a periodic pattern with distinct peaks and valleys. This behavior is indicative of the air conditioner’s operational cycle, which alternates between active cooling (high power consumption) and idle or maintenance states (low power consumption). The measured amplitude ranges from 0 to approximately 2500 watts, with each cycle reflecting the compressor’s on-off switching to maintain the desired room temperature.

1. Peak Power Consumption: The compressor operates at a peak power of 2500 W, consistent with its maximum cooling load.
2. Duty Cycle: The time interval for one complete on/off cycle is approximately 10 s, indicating the air conditioner’s operational frequency.

The periodic switching behavior evident in the power measurements corresponds to state transitions in the model. For example, a thermostat’s control logic might signal the system to switch from idle to active cooling based on temperature thresholds, which is reflected in the power measurement profile.

### Refrigerator

**Fig. 8:**
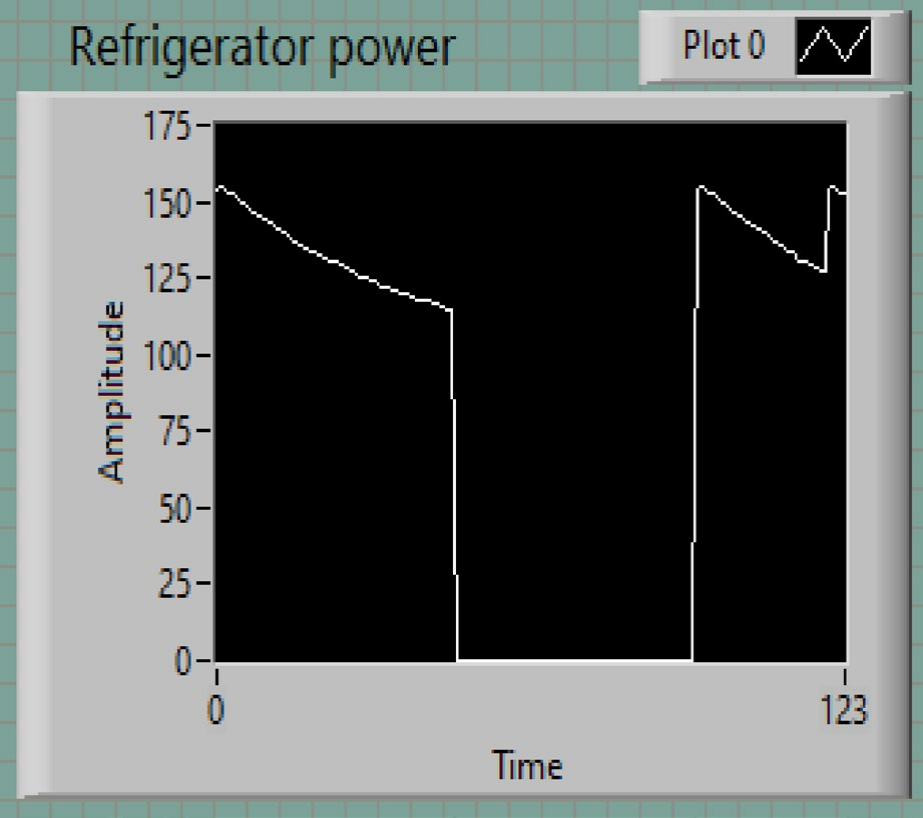
Refrigerator power.

The power consumption profile of a refrigerator, as shown in Fig. 8, illustrates a cyclical pattern characterized by intervals of active operation and idle phases. The measured amplitude ranges from 0 to approximately 150 watts. The gradual decrease in power during active phases followed by abrupt transitions to idle states corresponds to the compressor cycling on and off to regulate the internal temperature. The power measurement reflects the operational phases of a typical household refrigerator:

1. Initial Power Level: The power consumption begins at 150 W, reflecting the compressor’s startup load.
2. Decay Pattern: Over approximately 60 s, the power consumption reduces gradually to about 100–125 W, representing the reduced load as the interior temperature stabilizes.
3. Idle Period: The power periodically drops to 0 W for roughly 20 s, corresponding to the compressor being off.

This cyclic behavior is essential for maintaining the thermal balance inside the refrigerator while minimizing energy use.

### Battery behavior

**Fig. 9:**
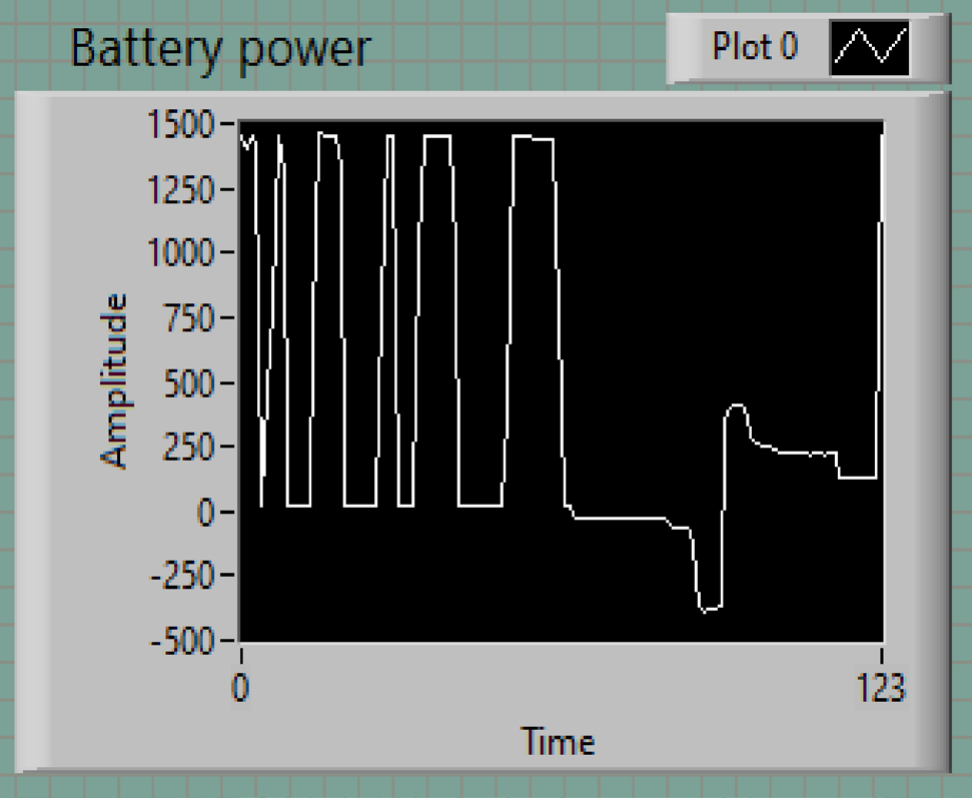
Battery power.

The battery profile, depicted in Fig. 9, shows distinct phases of charging and discharging, with amplitudes ranging from − 250 W (discharging) to 1500 W (charging). This dynamic operation is managed by a battery management scheme to balance energy supply and demand efficiently.

- Maximum Charging Power: The battery reaches a peak charging power of 1500 W, indicating its capability to store excess energy.
- Discharging Power: The discharging power reaches a minimum of −250 W, supplying energy to meet demand.
- Transition Points: There are observable transitions where charging ceases, and discharging begins, typically after approximately 20 s, demonstrating a time-dependent operation based on load conditions.

### PV power

**Fig. 10:**
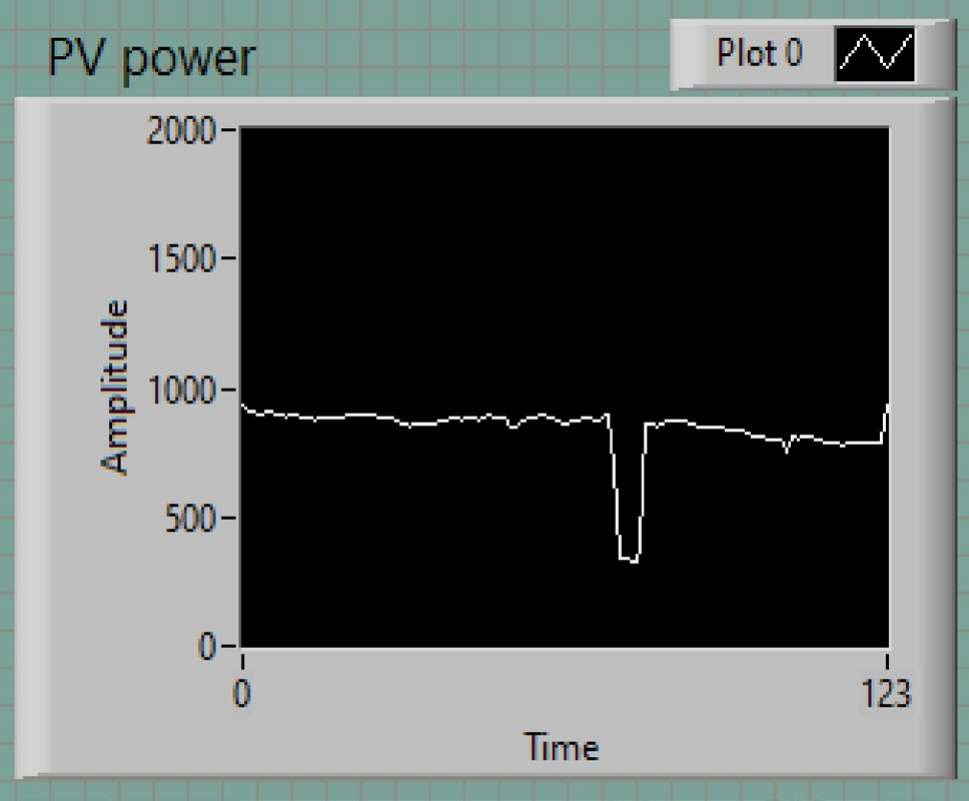
PV power.

The PV power output remains stable at approximately 1000 W for a duration of 10 min, reflecting the sustained solar potential under the conditions studied. However, temporary reductions in solar irradiance, likely caused by factors such as cloud coverage, dust accumulation, or other obstructions, lead to brief decreases in power generation lasting approximately 10 s, as shown in Fig. 10.

### Energy transactions

**Fig. 11:**
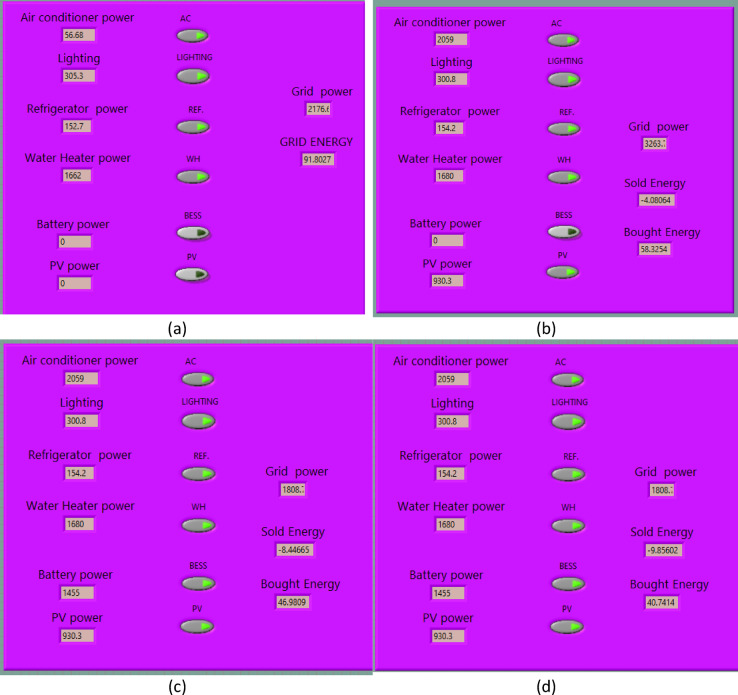
Energy transaction results: (**a**) default case, (**b**) home with PV, (**c**) home with PV/battery, (**d**) comfort zone case

This section presents the energy transaction behaviors recorded using the proposed prototype under various operating scenarios. These scenarios demonstrate the system’s ability to manage energy flows between the grid, PV panels, and the battery to achieve economic and operational benefits in a smart home environment. The importance of these observations lies in validating the effectiveness of the prototype in optimizing energy use and reducing dependency on grid energy. LabVIEW analysis shown in Fig. 11.

#### Scenario 1: default case (grid-only operation)

In this baseline scenario, the household operated solely on grid power without PV or battery support. LabVIEW recorded a total energy consumption of 91.8 Wh over the 10-minute test period. This value reflects the natural variations in power usage from active appliances during the observation window, without any energy management or optimization applied.

#### Scenario 2: grid with PV integration

Here, an 8 kW rooftop PV system was connected and active during the test. Based on real irradiance conditions at the time, the LabVIEW system measured a total grid import of 58 Wh and PV export of 4 Wh. These figures were calculated dynamically, not from assumptions or averages, and represent realistic solar participation under partially loaded system conditions.

#### Scenario 3: grid with PV and battery storage

In the third scenario, a battery was added to store excess PV energy instead of selling it to the grid. This configuration resulted in a grid energy consumption of 46.9 Wh, while 8.44 Wh of energy was sold back to the grid. The inclusion of a battery improves energy efficiency by enabling the storage of PV energy during peak generation times, which can then be utilized during periods of higher demand or reduced solar irradiance. This minimizes grid reliance and enhances the overall economic viability of the system.

#### Scenario 4: smart home energy management system

A SHEMS was employed, incorporating comfort zones and appliance scheduling to maximize economic benefits. This advanced setup reduced grid energy consumption to 40 Wh, while the energy sold to the grid increased to 9.85 Wh. The HEMS optimizes energy use by scheduling appliances to operate during times of high PV energy availability, reducing energy costs, and increasing revenue from energy sales to the grid.

The proposed prototype effectively demonstrates the potential of an integrated energy management approach in reducing grid dependency and improving economic outcomes. By leveraging PV systems, battery storage, and advanced load scheduling through SHEMS, the system maximizes the utilization of renewable energy while minimizing energy costs for households. These results highlight the need for implementing such prototypes in real-world applications, especially in developing countries, where efficient energy management is critical for sustainable development.

### Sustainability evaluation

#### Economic analysis

The economic evaluation presented in this study is based on short-term prototype operation due to laboratory limitations, such as constrained programmable load availability and dependence on actual solar irradiance. To ensure meaningful analysis, the results were extrapolated using previously validated theoretical models published in our earlier work^12^, which covered a full week of energy data for a 200 m² household. This approach allows for a realistic yet scientifically grounded estimation of cost savings and payback period, aligning the experimental setup with practical applications in developing regions. The methodology and theoretical parameters used to support this analysis are also provided in the supplementary material for reference.

In Egypt, recent regulations require homeowners to install rooftop PV systems covering at least 50% of the available rooftop area^16^. As a result, the cost of the PV system is considered a mandated baseline investment and was therefore excluded from the payback period calculation.

The validated theoretical model assumed a 200 m² residential household, representative of a medium-to-high consumption tier under Egypt’s IBR tariff scheme^12^. The baseline total annual electricity consumption was approximately 10,680 kWh, with the peak month (August) reaching around 1,320 kWh, mainly driven by intensive cooling demand. These baseline values provide the reference for the relative monthly savings (%) shown in Table 2, ensuring the reductions can be interpreted in relation to actual household demand and tariff classification.

The economic feasibility of three energy management strategies—PV-only, PV-battery, and comfort zone optimization—was assessed through prototype implementation.

*PV-Only system:* The PV-only configuration resulted in an estimated annual energy cost saving of 16,069.2 EGP (≈ $321 USD), yielding a payback period of 1.42 years. These findings are consistent with theoretical projections and support the financial viability of PV system investments under Egypt’s current electricity tariff scheme.

*PV-Battery system*: Incorporating battery storage into the PV system further improved energy utilization and reduced reliance on the national grid. The system achieved annual savings of 26,782 EGP (≈ $535.64 USD) with a payback period of only 0.85 years (≈ 10.25 months). This underscores the value of storage integration in enhancing economic returns.

*Comfort zone management*: This strategy involved optimizing appliance scheduling and temperature settings without the need for hardware upgrades.

It achieved the highest cost reduction, an 81% decrease in electricity bills (corresponding to 33,374.6 EGP annually), with an exceptionally short payback period of 0.68 years (≈ 8.2 months). The absence of capital investment makes this approach particularly attractive for widespread adoption, especially in resource-constrained settings. Tables 2 and 3 summarize the prototype estimated payback periods and component costs, respectively.

**Table 2:** Payback period for energy management strategies.

Scenario	Monthly saving (%)	Annual saving (L.E.)	Payback period (year)
PV only	39	16069.2	1.424
PV/battery	65	26,782	0.85411
Comfort zone	81	33374.6	0.6854

**Table 3:** Prototype hardware cost breakdown.

Item	Quantity	Cost (L.E.)/Item	Cost (L.E.)
Controller DAC 6002	1	11,560	11,560
Current transformer	8	577	4616
Voltage transformer	3	1697	5091
Auxiliary components	1	250	250
Contactor	2	504	1008
Relay	2	175	350
Total Cost (LE)			22,875

These findings emphasize that comfort zone management provides the most cost-effective solution, especially in high-tariff contexts, while PV-battery systems offer superior long-term returns through energy independence. The outcomes strongly support the deployment of such technologies in Egypt under IBR tariff structures, with the presented results specifically quantified for Greater Cairo households.

#### Social evaluation

Smart home applications enable real-time load optimization. Reported savings range between 15% and 30%, depending on technology type and behavioral patterns^17,18^. In Egypt, the average annual household electricity consumption is approximately 7,808 kWh/year^19,20^.

The conservative 20% reduction applied in this study is not derived from the prototype results but was intentionally chosen to reflect a cautious estimate. The prototype experiments showed higher savings potential, yet such outcomes depend on optimal user behavior and controlled conditions. To avoid overestimating societal benefits and to provide a robust baseline for policymaking, we adopted the 20% reduction figure as a conservative benchmark, aligned with international studies on average household savings from SHEMS^17,18^.

It is also clarified that the figure of 7,808 kWh/year represents the *national average household electricity consumption* reported in Egypt’s official statistics, not the consumption of a 200 m² household. The household size (200 m²) was mentioned only as a representative example and should not be directly correlated with the national average consumption.

For a 200 m² household, assuming a conservative 20% reduction:

$$E_{{smart}} = {\text{ }}7,808{\text{ }} \times {\text{ }}0.20{\text{ }} = {\text{ }}1,561.6{\text{ }}kWh/year$$

Thus, a potential saving of 1,561.6 kWh/year per household is expected with smart home technology adoption. According to Egypt’s national statistics, high-income households (annual income of EGP 144,000–180,000) represent approximately 10% of the population in Greater Cairo^21^. Cairo’s total population is about 23 million^22^, with an average household size of 4 persons^23^.

Thus, the estimated number of high-income households is:

$$High - income{\text{ }}households = ~\frac{{23,000,000}}{4}x~0.1 = 575,000$$

Although 70% of urban residents are aware of energy-efficient technologies, only 40% have adopted them due to initial cost barriers^24^. Therefore, socioeconomic disparity and awareness levels play critical roles in determining the actual penetration and effectiveness of these technologies.

The societal energy savings achievable by adopting smart home systems in 575,000 households:

Full-scale implementation of SHEMS among high-income households in Greater Cairo can reduce electricity consumption by approximately 0.9 TWh annually. An 8 kWp rooftop PV system, under Cairo’s solar irradiation of 1,900 kWh/kWp/year^25^, yields:

$$E_{{PV}} = {\text{ }}8{\text{ }} \times {\text{ }}1,900{\text{ }} = {\text{ }}15,200{\text{ }}kWh/year$$

Assuming 70% self-consumption:

Thus, a high-income household with PV can offset up to 10,640 kWh annually.

Total societal savings, assuming universal PV adoption among high-income households:

$$E_{{total,{\text{ }}PV}} = {\text{ }}575,000 \times 10,640 = {\text{ }}6.11TWh/year$$

Such adoption could reduce Greater Cairo’s grid demand by 6.11 TWh/year, contributing significantly to urban energy resilience and sustainability targets. All aggregated results presented here refer **only to Greater **Cairo (575,000 high-income households).

The quantitative findings show that integrating SHEMS with rooftop PV adoption among high-income households in Greater Cairo can yield ≈ 0.90 TWh/year in efficiency-driven savings and ≈ 6.12 TWh/year in PV-driven offsets. Together, these measures significantly enhance urban energy resilience, reduce grid dependency, and align with Egypt’s sustainability and energy diversification goals.

#### Environmental impact

Using an updated grid CO₂ emission factor of 0.806 kg CO₂/kWh[26], the per-household reduction from the conservative smart-home saving of 1,561.6 kWh/year is 1,258.65 kg CO₂/year. Assuming 10% adoption of SHEMS in Greater Cairo (≈ 575,000 households), the aggregate annual CO₂ reduction from smart-home measures is ≈ 723,724 ton CO₂/year.

An 8 kWp rooftop PV system in Greater Cairo yields approximately 15,200 kWh/year (PVGIS). Applying the same emission factor gives ≈ 12,251.2 kg CO₂/year avoided per PV household. For 1% rooftop PV adoption in Greater Cairo (≈ 57,500 households) — reflecting building rooftop availability (average building footprint ≈ 200 m², ~ 5 floors, ~ 50% usable roof) — the aggregate annual CO₂ reduction from PV self-consumption is ≈ 704,444 ton CO₂/year.

Combining both measures under the above Greater Cairo assumptions produces an estimated total avoided emission of ≈ 1,428,168 ton CO₂/year (≈ 1.43 Mton CO₂/year). These figures are reported for Greater Cairo only and keep the environmental assessment consistent with the economic and social analyses presented elsewhere in the manuscript.

The integrated economic, social, and environmental assessments thus demonstrate that targeted adoption of SHEMS and rooftop PV in Greater Cairo can produce meaningful urban-scale CO₂ reductions while guiding city-level policy and incentive design.

## Conclusion

This study experimentally validated a cost-effective Smart Home Energy Management System (SHEMS) prototype tailored for residential consumers under Egypt’s IBR tariff. The system successfully integrated PV generation, battery storage, and comfort zone-based load scheduling, yielding measurable gains in energy efficiency, affordability, and environmental performance. Notably, the PV-battery combination achieved the highest long-term savings, while comfort zone-based scheduling offered a no-cost behavioral approach that incrementally improved energy performance without requiring hardware upgrades.

Although the system aligns with global sustainability goals, particularly SDGs 7 (Affordable and Clean Energy), 11 (Sustainable Cities and Communities), and 13 (Climate Action), certain limitations merit further exploration. These include the effects of seasonal solar variability, long-term battery degradation, and behavioral unpredictability among users. Additionally, while comfort zone management was tested in conjunction with other system components, its standalone performance was not separately quantified and will be evaluated in future work.

Future research will focus on:

- Quantifying system performance across multiple climatic seasons;
- Incorporating battery aging models into control strategies;
- Testing comfort zone scheduling as a standalone method;
- Expanding the prototype’s adaptability to other developing countries by incorporating region-specific technical, economic, and policy data.

## Supplementary Information

Below is the link to the electronic supplementary material.

Supplementary Material 1

## Data Availability

The datasets used and generated during the current study are available from the corresponding author upon reasonable request.

## Abbreviations

SHEMS: Smart home energy management systems
IBR: Inclining block rate
PV: Photovoltaic
TOU: Time-of-use
RTP: Real time pricing
HVAC: Heating, ventilation, and air conditioning
SOC: State of charge
MILP: Mixed-integer linear programming
AI: Artificial intelligence
GIS: Geographic information system
UIS: User interface screen
