# Supplementary material for: Smart home energy management for sustainable socioeconomic development in Egyptian households
Source: Sci Rep. 2026 Feb 6;16:5654. doi: 10.1038/s41598-026-35705-0 (PMC12891580; doi:10.1038/s41598-026-35705-0)
Supplement: Supplementary file 1 — Supplementary Material 1 [file 41598_2026_35705_MOESM1_ESM.docx]

1. ***Air*** ***Conditioner*** **(AC)**

〖T_i ,T〗_(i+1): Room temperature in time intervals i and i+1 ;

∆t :Length of time intervals ;

C_HVAC: Cooling/heating capacity, positive for heating and negative for cooling (Btu/h);

∆c :Energy needed to change the room air temperature, which can be calculated as the product of the specific heat capacity of air and the house volume .

G_i: Heat gain rate of the house during time interval, positive value results in an increase in room temperature; and negative value results in a decrease in room temperature (Btu/h). It can be represent mathematically, as follows [35];

$G_{i}=\left( \frac{A_{wall}}{R_{wall}}+\frac{A_{cieling}}{R_{ceiling}}+\frac{A_{window}}{R_{window}}+\frac{11.77 Btu}{Fx {Ft}^{3}}.n_{ac}.V_{house} \right)+SHGC.A_{window\_south}.H_{solar}.\frac{3.412 \frac{Btu}{Wh}}{10.76\frac{{ft}^{2}}{m^{2}}}+H_{p}$ (10)

Where , A_wall, A_cieling, A_window, and represent the area of the wall, ceiling, and window of the house, and R_wall, R_ceiling , R_window represent the heat resistance of the wall, ceiling, and window,

n_ac: number of air changes in each time interval (1/h),

V_house: house volume,

SHGC: Solar heat gain coefficient of windows,

H_solar: solar radiation heat power

H_p: heat gain from people (Btu/h).

|  | AC-1 | AC-2 | AC-3 | AC-4 |
| --- | --- | --- | --- | --- |
| A_wall | 2646 | 2646 | 2646 | 2646 |
| R_wall | 11 | 11 | 11 | 11 |
| A_cieling | 517 | 517 | 820 | 410 |
| R_ceiling | 30 | 30 | 30 | 30 |
| A_window | 130 | 130 | 175 | 130 |
| R_window | 30 | 30 | 30 | 30 |
| $n_{ac}$ | 0.01 | 0.01 | 0.01 | 0.01 |
| $V_{house}$ | 6004 | 6004 | 8050 | 2080 |
| $SHGC$ | 0.32 | 0.32 | 0.32 | 0.32 |
| $A_{window\_south}$ | 130 | 130 | 130 | 130 |
| $H_{solar}$ | 100 | 100 | 100 | 100 |
| $H_{p}$ | 245 | 245 | 245 | 245 |
| Cair | 0.0195 | 0.0195 | 0.0195 | 0.0195 |
| P | 5000 | 5000 | 8000 | 3000 |

1. ***Electric Oven [36]***

*C_e_, C_in_ and C_a_: thermal mass of heating element, oven cavity and oven external parts (J/ ◦C), T_e_, T_air_, T_in_ and T_a_: temperature of heating element, air outside oven, inside oven and external parts (◦C), R_ei_, R_ia_ and R_ia_: thermal resistance between heating element and cavity, externals and cavity and surroundings and cavity (W / ◦C), P: the electric power extracted.*

| *C_e_* | *112* |
| --- | --- |
| *C_in_* | *3690* |
| *C_a_* | *4590* |
| *T_air_* | *28* |
| *R_ei_* | *0.228* |
| *R_ia_* | *0.181* |
| *R_a_* | *0.252* |
| *P* | *2400* |

1. ***Refrigerator [37]***

*C_e_, C_c_ - thermal mass of evaporator and cooling cavity (J/◦C), Tc, T_air_ and T_e_ - temperature inside the cooling space, ambient and evaporator (◦C), Rec, R_ec_ - thermal resistance between evaporator and cooler, between surroundings and cooler (W /◦C), COP - coefficient of performance, P: extracted the electric power.*

| *C_e_* | *0.64* |
| --- | --- |
| *C_c_* | *3.2* |
| *T_air_* | *32* |
| *R_ec_* | *0.325* |
| *R_i_* | *0.475* |
| *COP* | *0.9* |
| *P* | *1200* |

1. ***Domestic iron [36]***

*C_m_: thermal mass of heating element (J/ ◦C); T_T_, T_a_: temperature of heater/iron, ambient (◦C); R_A_: thermal resistance between element and surroundings and cooler (W / ◦C), P: the electric power.*

| *C_m_* | *1030.4* |
| --- | --- |
| *T_a_* | *28* |
| *R_A_* | *0.3077* |
| *P* | *1300* |

1. ***Dishwasher [37]***

The dishwasher daily cycle can be divided into three processes: wash, rinse, and dry. Dish washer consumed power varies during each process. On average, dishwasher complete operating cycle consumes about 1.44 kWh for 105 minutes. Daily whole dishwasher operating cycle can be shifted to limit home peak load power.

1. ***Washing machine [37]***

Cloth washing machine cycle has three main stages: wash, rinse, and spin. On average, it takes about 45 minutes to complete one operating cycle. There is a drying additional optional process. Although relative hot sunny climate is available during most of the year in Egypt, there is a growing increase of the washing machine with a dryer in the Egyptian market. Drying process takes about 60 minutes with relatively high-power consuming compared to other washing process.

For instance, main cycle process load power varies from 0.52 to 0.65 kW. While, drying process power, for the same case, is about 2.97 kW.

1. ***Photovoltaic Panel PV [30-33]***

The total developed power from PV system calculated from the following formula [30-33].

$P_{PV}=G.G_{T}.f_{PV}.Y_{PV}$ (1)

| *Y_PV_* | *= the rated capacity of the PV array, meaning its power output under*[*standard test conditions*](https://www.homerenergy.com/products/pro/docs/latest/standard_test_conditions.html)*[kW]* |
| --- | --- |

*Where:*

*f_PV_= the PV derating factor [%]*

*G= the solar radiation incident on the PV array in the current time step [kW/m2]*

| PV parameters | |  |  |  |  |  |  |  |
| --- | --- | --- | --- | --- | --- | --- | --- | --- |
| Y_PV_ | = the rated capacity of the PV array, meaning its power output under standard test conditions [kW] | | | | | | | 8 |
| f_PV_ | = the PV derating factor [%] |  |  |  |  |  |  | 45 |
| G_t_ | = the incident radiation at standard test conditions [1 kW/m2] | | | |  |  |  | 1 |

*G_T_ = the incident radiation at standard test conditions [1 kW/m2]*

***H- Battery[34]***

Battery models and constraints can be formulated as follows:[34]

$P_{ESS}\left( t \right)=P_{ESS}^{ch}\left( t \right)u_{ESS}\left( t \right)+P_{ESS}^{disch}(t)(1-u_{ESS}\left( t \right))$ (2)

$C_{ESS}\left( t \right)=C_{ESS}\left( t-1 \right)+P_{ESS}^{ch}\left( t \right)u_{ESS}\left( t \right)\Delta t\alpha_{ESS}^{ch}+P_{ESS}^{disch}\left( t \right)\left( 1-u_{ESS}\left( t \right) \right)t/\alpha_{ESS}^{disch}$ (3)

$C_{ESS}^{int}=C_{ESS}\left( t-1 \right) t=1$ (4)

Where:

$C_{ESS}$ ESS capacity (kWh)

$P_{ESS}^{ch}$ ESS charging power (kW)

$P_{ESS}^{disch}$ ESS discharging power (kW), PESS dis < 0

$\alpha_{ESS}^{ch}$ ESS charging efficiency

$\alpha_{ESS}^{disch}$ESS discharging efficiency

$C_{ESS}^{int}$ ESS initial capacity (kWh)

$u_{ESS}$ Binary variable, the charging/discharging state of ESS, 1 if ESS charging, else 0

| Min. Ch (%) | 20 |
| --- | --- |
| Max. Ch (%) | 90 |
| Capacity (kWh) | 30 |
